# Association between superstition levels on sports-specific success motivation in active athletes: a study in Ankara Province

**DOI:** 10.55730/1300-0144.6189

**Published:** 2024-07-25

**Authors:** Canan SAYIN TEMUR

**Affiliations:** Department of Sports Management, Faculty of Sport Sciences, Ankara Yıldırım Beyazıt University, Ankara, Turkiye

**Keywords:** Sports, superstition, motivation, success motivation

## Abstract

**Background/aim:**

When athletes perceive themselves as strong, they may seek to maintain their motivation through the control of random external factors. The present study examines the effect of superstitions on sports-specific success motivation among active athletes.

**Materials and methods:**

The research population consisted of licensed athletes in the athletics branch, while the sample comprised licensed and active athletes based in Ankara. A total of 408 athletes participated in the research, including 201 women, and 207 men. The participants completed a three-part questionnaire consisting of a Personal Information Form, the Motivation in Sports Scale, and the Superstitious Beliefs and Behaviors in Sports Scale. The obtained data were analyzed using IBM SPSS Statistics, version 22.0.

**Results:**

It was concluded from the study that the superstition levels of active athletes had a positive effect on sports-specific success motivation.

**Conclusion:**

Superstition levels among the respondent active athletes were positively associated with sports-specific success motivation. The findings of the present study suggest that understanding and encouraging superstitious practices among athletes may contribute to enhancing the confidence and motivation of athletes, and consequently, athletic performance.

## 1. Introduction

Actions, behaviors, or beliefs that have no logical basis, stem from ignorance or misinterpretation of causality, and are believed to circumvent natural laws are called superstitions. People continue to be influenced by superstitions with origins in cultural beliefs from millennia ago. Human curiosity about life’s mysteries, the desire to overcome difficulties, the expectation of better outcomes from various situations, and the hope of reversing feelings of helplessness form the basis of superstitions in individuals [1]. Some individuals act on their beliefs and engage in specific practices with the expectation that doing so will reduce or enhance the impact of an event or situation that may or may not occur [2]. Superstitious beliefs and behaviors are more likely to be observed during economic crises and cultural dissolution, and may be more prevalent in some fields than in others [3]. Sport is a field in which superstitious behaviors are commonly observed. A broad spectrum of superstitious behaviors has been observed in sport at all levels, from amateur to professional. Many top athletes in various disciplines are known to be inclined toward superstitious behaviors. For example, tennis players who always wear the same wristband in the belief that it contributes to their success; football players who perform a specific pregame ritual when entering the field or cut the toes off their socks; and basketball players who habitually touch their shoes. These behaviors are rooted in the fear of losing and the desire to win in individuals, and have prompted numerous studies of the superstitions held by athletes and the factors affecting them [4–8]. Studies have identified superstitions as one of the factors motivating athletes and contributing to their success. Training and performance tests, along with muscle strength, contribute significantly to athletic success [9]. Aside from physical strength and performance, the contributions of mental strength and motivation to success should not be ignored. The present study analyzes the extent to which superstitions affect motivation and success through an examination of the effect of superstition levels on sports-specific success motivation in active athletes.

### 1.1. Superstition

Throughout history, people have engaged in culturally rooted actions that they believe can turn uncontrollable situations in their favor, despite having no objective basis in reality [10]. Such behaviors, known as superstitions, are widely accepted around the world [11]. Fears related to life and death, economic and worldly hardships, and coping with pain and illness all lead individuals to engage in superstitious behaviors [12].

The concepts of superstition and myth have long been debated, and continue to challenge researchers. The term “superstition” is derived from the Arabic word *“butlan”*. Whether religious or not, accepting a system as it is can also be expressed as belief [13]. Budak defines superstition as “the belief that prayer, magic, black magic, wearing objects believed to bring luck, writing talismans, summoning spirits, etc., can forcibly alter outcomes” [14]. From birth, individuals develop a desire to know the future and understand natural laws, and superstitions arise from the desire to predict one’s future [15]. People who engage in superstitious behaviors generally do so to minimize or maximize the impact of future events they fear might occur [3].

There is no universally accepted definition of superstition that is accepted by all societies. While some consider them to be meaningless and empty beliefs and practices, others perceive them as culturally appropriate and meaningful [16]. In general, superstition is defined as the belief that events and conditions outside one’s control can affect outcomes [17].

#### Motivation

Motivation is the force that drives a person to perform a specific action in a particular situation. In the context of this study, motivation is conceptualized as the internal and external processes that initiate, direct, and sustain goal-oriented behaviors. Understanding personal motivational processes is crucial, as they compel individuals to consider their goals, strive to achieve their targets, and maintain desired behaviors over time [18]. Understanding personal motivational processes is crucial, as they compel people to consider their goals, to attain their personal targets, and to understand the conditions necessary to maintain their desired behaviors. The concept of motivation encompasses the perceived emotions related to the task and the desire to bring it to conclusion. Motivation can be both inhibiting and driving [19].

The source of motivation is defined as the need that drives an individual to take action. These needs are not limited to physiological deficiencies; they also include ongoing psychological needs that continuously influence behavior [20].

#### Superstitions and motivation among athletes

Athletes may perceive themselves as strong, but may draw on their superstitious beliefs to continuously and technically increase their performance through repeated attempts to control different external factors [8]. These repeated actions and superstitions, commonly referred to as rituals, differ from the athletic preparations ahead of competitions, and it may be difficult for outsiders to understand their importance to the athlete. While these actions may have no direct impact before a competition, they may positively affect performance as part of the athlete’s mental preparation. Therefore, mental preparation before the competition is a process that is developed and refined over time through experience and practice. The behavioral and cognitive strategies adopted by individuals when exercising to increase performance have been investigated in previous studies in the literature [21,22].

Studies analyzing the relationship between sport and superstitions have addressed a wide range of disciplines, including basketball [23], tennis [24], swimming [25], ice hockey [26], baseball [27], and volleyball [28], underscoring the relevance of the present study.

The primary motivators in sports are the coach and the spectators. Coaches play a crucial role in encouraging athletes to perform at their best, whether through praise or punishment. Praise can promote positive feelings in the athlete toward the coach, competitions, and themselves, and can promote appropriate behaviors, while punishment and focusing on mistakes can hinder the maintenance of positive feelings. Appropriate rewards instill confidence in athletes and can contribute to their success; while punishment can lead to insecurity and confusion [29]. The present study analyzes the effect of the superstition levels in active athletes on sports-specific success motivation.

## 2. Materials and methods

### 2.1. Population and sample

The research population consists of all licensed athletes aged 18 and above in Ankara, Türkiye who were active in the athletics branch in 2022 (n=6388). The open-source OpenEpi software was used to calculate the appropriate sample size. Based on a 95% confidence level, a minimum of 369 individuals was required from the population of licensed and active athletes in Ankara. The sample group was selected using a convenience sampling method. Ultimately, a total of 408 athletes (201 women, 207 men) participated in the research, taking part on a voluntary basis. Ethical approval for the study was obtained from the Ankara Yıldırım Beyazıt University Faculty of Medicine Ethics Committee, protocol number (2022-911).

### 2.2. Data collection tools

The respondents completed a Personal Information Form, the Sports-Specific Success Motivation Scale (SSSMS), and the Superstitious Beliefs and Behaviors Inventory in Sports.

The Personal Information Form was designed by the researcher following an extensive review of the literature, and aimed to garner data on the sociodemographic characteristics of the respondents that are believed to influence their beliefs, superstitious thoughts, and success motivation. The form included questions to obtain data on the sex, age, educational status, branch, active years in sports, participation in the national team, number of times participating in the national team, and the importance of sporting success of the respondents.

The SSSMS was developed by Willis in 1982 and adapted to Turkish by Tiryaki and Gödelek in 1997. The scale comprises 40 items under three subscales: Power Display Motive (PDM) (12 items), Success Approach Motive (SAM) (17 items), and Failure Avoidance Motive (FAM) (11 items). The scale includes items reflecting individual judgments as well as positive and negative feelings related to different aspects of sport participation [30].

The Superstitious Beliefs and Behaviors Inventory in Sports, a key component of our study, was developed by Buhrman et al. in 1982 [31], commonly referred to in the literature as the “Superstitious Ritual Questionnaire (SRQ)”, and was adapted to Turkish by Barut in 2008 [32]. This inventory comprises six categories and provides valuable insights into the superstitious behaviors of athletes, including those related to clothing and appearance, lucky objects, pre- and during-game rituals, in-game behaviors, team superstitions, and prayer.

### 2.3. Data analysis

Data were analyzed using IBM SPSS Statistics, version 22.0 (IBM Corp., Armonk, NY, USA). The normality of the variables was assessed using the Shapiro-Wilk test and through an examination of skewness and kurtosis values divided by their standard errors. Both the scale and its subdimensions met these two criteria, and so the normal distribution assumption was satisfied. Based on the above, parametric tests were employed. An independent sample t-test and a one-way analysis of variance (ANOVA) were used to determine the relationships between socio-demographic variables, superstitions and behaviors in sports, and sports-specific motivations. Pearson’s correlation test was conducted to determine the relationship between superstitions and behaviors in sports and sports-specific motivation. Cronbach’s alpha internal consistency coefficients were calculated for both scales, calculated as 0.874 for the SSSMS, and 0.922 for the SRQ. Both values were within statistically acceptable limits. A p-value of <0.05 was set for all statistical tests.

## 3. Results

The number and percentage distributions of the respondents’ sociodemographic data are presented in Table 1.

A total of 408 people participated in the study (49.3% women, 50.7% men); 33.1% were aged 18–20 years, 17.2% were aged 21–23 years, 18.9% were aged 27–29 years, and 16.4% were 29 years and older. As regards to educational attainment, 4.9% had completed primary school, 17.4% had completed high school or equivalent, 61.3% held undergraduate degrees, and 16.4% held postgraduate degrees. Concerning the primary athletic discipline of interest, 72.5% competed in running events, 11.3% in throwing events, and 16.2% in jumping events. Furthermore, 9.6% had been actively involved in sports for 1–3 years, 12.7% for 3–5 years, 22.7% for 5–7 years, 26.7% for 7–10 years, and 28.2% for over 10 years; and 43.4% had participated in the national team. Among those who had participated in the national team, 26.2% had participated 1–10 times, 13.5% had participated 10–20 times, and 3.7% had participated 20–30 times. When asked about the importance they placed in sports, 2.7% considered sporting success to be of minor importance, 20.1% considered it important, and 77.2% considered it very important.

The study aimed to determine the relationship between sex, age, educational status, branch, years in active sport, participation in the national team, number of participations in the national team, importance of sporting success, and motivations and superstitions related to sporting success. The data obtained from the analyses are presented in Tables 2–10.

The difference between the general total scores and subdimension scores from the SSSMS and the SRQ were analyzed with respect to sex using an independent samples t-test, revealing no statistically significant difference between the mean general total scores of the male and female respondents. In contrast, a significant difference was observed between the male and female respondents in the SAM and FAM subscales of the SSSMS, and the Pre- and During-Game Rituals subscales of the SRQ, with women scoring higher in all these subscales. The difference between the general total scores and total subdimension scores of the SSSMS and SRQ with respect to age was examined with a One-Way Analysis of Variance (ANOVA), and a significant difference was observed between the general total scores and subscales of both scales in regards to age, being higher in the 27–29 age group in the SSSMS and higher in the 21–23 age group in the SRQ. Significant differences were also observed with respect to age in the PDM and SAM subscales of the SSSMS and the Clothing and Appearance, Lucky Objects, Pre-Game Rituals, and Praying subscales of the SRQ. Among the SRQ subscales, the 18–20 age group scored higher than the other age groups in the Clothing and Appearance subscale; the 21–23 age group scored higher in the Lucky Objects and During-Game Rituals subscales; and the 29 years and older age group scored higher in the Praying subscale. The 24–26 years age group scored higher than the other groups in the PDM subscale of the SSSMS, and the 29 years and older age group scored higher in the SAM subscale.

The difference between the general total scores and subdimension scores of the SSSMS and the SRQ with respect to educational status was examined with a one-way ANOVA. The analysis revealed a significant difference between the general total scores of both scales and all subscales other than in the Pre-Game Ritual subscale. High school and equivalent graduates scored highest in the general total score and in the Clothing and Appearance, Lucky Objects, and During-Game Rituals subscales in the SRQ. Those with postgraduate degrees had the highest general total scores and scored highest in the SAM and FAM subscales of the SSSMS, as well as in the Praying and Team Behaviors subscales of the SRQ. Those with undergraduate degrees, on the other hand, scored highest in the PDM subscale of the SSSMS.

The difference between the general total scores and subdimension total scores from the SSSMS and the SRQ in regards to the primary sporting branch variable was examined with a one-way ANOVA. No significant difference between the PDM and FAM subscales of the SSSMS and the Praying subscale of the SRQ were observed with respect to the branch variable. In contrast, a significant difference was observed between the general total scores of both scales and the SAM subscale of the SSSMS and the general total scores of the SRQ, and between the Clothing and Appearance, Lucky Objects, Pre- and During-Game Rituals, and Team Behaviors subscales and the branch variable. The respondents in the running disciplines scored higher scores in both the total scores and the subscale scores than those participating in throwing and jumping events.

The difference between the general total scores and subdimension total scores from the SSSMS and the SRQ in regards to the years of sporting activity variable was examined with a one-way ANOVA. No significant difference was observed between the general total scores of the SRQ and the Clothing and Appearance, Lucky Objects, Pre- and During-Game Rituals, and Praying subscales in terms of the years of sporting activity variable. In contrast, a significant difference was observed between the general total SSSMS scores and all its subscales, as well as the Team Behaviors subscale of the SRQ, in regards to the years of sporting activity variable. The respondents who had been actively engaged in sports for 10 years and over scored higher in the general total score and the SAM subscale of the SSSMS and the Team Behaviors subscale of the SRQ. Those who had been actively involved in sports for 7–10 years scored higher in the PDM and FAM subscales of the SSSMS.

The difference between the general total scores and subdimension total scores in the SSSMS and the SRQ related to the national team participation variable was examined with an independent samples t-test. No significant difference was observed between the general total score from the SRQ, or the Clothing and Appearance, Lucky Objects, Pre-Game, Team Behaviors, and Praying subscales as regards to participation in the national team. In contrast, a significant difference was observed between the general total score and all subscales of the SSSMS and the During-Game Rituals subscale of the SRQ related to participation in the national team. The respondents who had participated in the national team scored higher in the general total score and all subscales of the SSSMS, while those who had not participated in the national team scored higher in the During-Game Rituals subscale of the SRQ.

The difference between the general total scores and subdimension total scores of the SSSMS and the SRQ and the number of national team participants was examined with a one-way ANOVA. The analysis revealed no significant difference between the Praying and Lucky Objects subscales of the SRQ and the number of participations in the national team variable. In contrast, a significant difference was observed between the number of participations in the national team variable and the general total score and all subscales of the SSSMS, as well as the general total score and the Clothing and Appearance, Pre- and During-Game Rituals, and Team Behaviors subscales of the SRQ. The respondents who had participated in the national team 10–20 times scored higher in all total scores and subscales than the other groups.

The difference between the importance of sports success for individuals variable and the general total scores and subdimension total scores of both the SSSMS and the SRQ was examined with a one-way ANOVA. The analysis revealed a significant difference between the general total score and all subscales of the SSSMS and the general total score and all subscales of the SRQ and the importance of sports success for individuals variable. The respondents who considered sporting success to be very important scored higher in all the total scores and subscales than the other groups.

Finally, the analysis revealed a statistically significant, very weak positive relationship between the general total score of the SSSMS and the general total score of the SRQ(r = − 0.020; p >0.05).

## 4. Conclusion and Discussion

In this study examining the effect of superstition levels on the sports-specific success motivation of active athletes, no significant relationship was observed between sports-specific motivation and sex, which is supported by other studies in the literature [33].

In addition to the general score of the SSSMS, no significant relationship was found between sex and the PDM subscale, while a significant relationship was found between sex and the SAM and FAM subscales, with women scoring higher in SAM and FAM. Similar high SAM levels were reported in a study of young female tennis players [33]. In another study in which men were reported to have higher PDM levels, a significant relationship was observed between sports-specific motivation and sex in the PDM and SAM subscales, but no such relationship with the FAM subscale [34]. However, other studies have reported higher PDM levels in women [35,36]. The variation in these findings is thought to be attributable to the traditional roles of the sexes in society, the responses of the participants to these roles, and their approach to competition. Similarly, women were reported to have higher SAM levels than men in another study [37].

Furthermore, significant relationships were observed between sports-specific motivation and age, educational status, branch, active sports years, participation in the national team, the number of participations in the national team, and the importance of sports success for individuals.

In examining superstitions in sports, the study found no significant relationship between superstitions and sex, active sports years, and participation in the national team. In contrast, a significant relationship was found between superstitions and age, educational status, branch, the number of participations in the national team, and the importance of sports success. Although no significant relationship was found between sex and superstitions in the overall scale score, male athletes were found to have higher SAM levels than female athletes, and this difference was attributed to cultural characteristics and expectations and the different roles assigned to each sex. Traditionally, men are expected to play more assertive roles than women [38]. In terms of failure avoidance, higher FAM levels were observed in female athletes than in male athletes [39]. Both general [40] sports-specific studies [31,41,32] have reported that women demonstrate more superstitious behaviors than men. Earlier studies have reported that women generally have higher levels of stress and anxiety than men [42], which explains their greater inclination toward superstitions.

There was no difference between the male and female respondents and lucky objects in the present study, and this is consistent with earlier studies in the literature [31,39,43]. Pre-game and in-game rituals were more common among the male respondents. Athletes engage in pre-game superstitious behaviors in the hope of influencing game performance [44], and this was more common among the male respondents of the present study, and aligned with the literature. In a previous study, pre-game superstitions were reported to be related more strongly to the athlete’s professional status than sex [45], with professional athletes being more likely to engage in pre-game rituals [10]. No difference was noted between the sexes in the team behavior and prayer subdimensions. Prayer and superstitions can instill in the athlete a sense of control over uncontrollable situations, helping them to regulate their mood and enhance their athletic performance [11]. Athletes pray to communicate with the Creator rather than for satisfaction, and so, realistically, prayer may also help them accept poor outcomes [46].

With regards to the age variable, there is a common belief that older individuals are more inclined towards superstitions, although it has been hypothesized [47] that young people are more superstitious due to their social concerns as they have less social interest and experience and are easily influenced by their social environment. A number of studies support Torgler’s argument that younger individuals tend to exhibit higher levels of superstition, which may be attributed to social uncertainty, limited life experience, and increased sensitivity to social influences. For example, in a study analyzing superstitious habits among students, a significant difference was observed in those related to clothing and appearance between athletes aged 20 and under and those in the other age groups [46]. This finding measures the impact of such superstitions and behaviors on sporting performance [31]. Athletes aged 21–23 scored higher than the other age groups in terms of their belief in lucky objects. Prayer was found to be more prominent among athletes aged 29 and older when compared to the younger age groups. Prayer and superstitions can provide individuals with a sense of control over uncontrollable situations, and so may help regulate mood and enhance athletic performance. The impact on performance was found to have an effect size of 0.25 [48]. Athletes may engage in prayer not merely for personal gratification but as a means of connecting with a higher power; in this sense, prayer can also facilitate the acceptance of unfavorable outcomes [49,50].

No significant difference in personality traits was noted among athletes in a Master’s thesis on athletes [51]. In terms of education, superstitions related to clothing and appearance were more common among individuals who had completed high school or equivalent than those with other educational attainment levels. Similarly, superstitions related to lucky objects were more common among those who had completed high school and equivalent when compared to the other educational attainment groups. Research assumptions on the relationship between education and superstitions generally report that the tendency towards superstitions decreases as the education level increases. For example, in their study on the relationship between superstitions and education level, Laura Otis and James Alcock concluded that university professors were less superstitious than students [52]. Accordingly, the assumption that individuals with lower education levels are more inclined towards superstitions than those with higher education levels has been confirmed [53]. A study of ice hockey players found that superstitious behaviors decreased as the duration of sporting activity increased [45]. As the time spent engaged in active sports increases year-on-year, self-efficacy scores increase, and the frequency of superstitious behaviors decreases [41]. This could be attributable to the strong relationship between time spent exercising and the frequency of superstitious behaviors, which aligns with Bandura’s theory on self-efficacy [54]. National athletes are more eager to achieve success, and more prominently seek to avoid failure. There is no relationship between participation in the national team and superstitions and behaviors. In the literature, some studies support this finding [55], as well as studies that do not show a significant difference in the subdimensions of imagery according to sports age [56].

Finally, the relationship between the general total scores obtained from the SSSMS and SRQ was examined to determine the effect of superstition levels on sports-specific success motivation in active athletes. The study revealed a very weak positive relationship between the general total scores of the two scales, which indicates that the superstition levels of active athletes have a weak but positive effect on sports-specific success motivation.

Superstitions and behaviors are prevalent among athletes in almost every sporting discipline. They are used by athletes to gain the sense of control necessary for success, and are an attempt to influence existing realities, adjust expectations, and find meaning in events. By targeting their inner world through superstitions, athletes can influence their motivation, emotions, and mental representations [57]. Previous studies have analyzed the superstitions and motivations of athletes separately [58–60]. In contrast, only a few have directly examined the effect of superstitions on sports-specific motivation in athletes. In one study analyzing the effect of superstition levels on success motivation in athletes engaged in individual and team sports, a significant positive relationship was observed between superstitions and behaviors in sports and sports-specific motivation levels [61], similar to the present study. While there are a number of studies in the literature explaining the effect of superstitions on sports motivation based on anxiety levels or success [5,62,63], the contribution to the literature of the present study lies in its direct examination of the effect of superstitions and behaviors on sports-specific motivation.

The use of superstitions and rituals by athletes before and during competitions is common, and contributes to their motivation. They are an integral part of sporting life, and when used correctly and appropriately, can motivate athletes during their preparations for competition, thereby contributing to success. It is believed that sports clubs, coaches, and athletic training centers can make a difference by encouraging such behaviors to motivate athletes, thus contributing to their success.

## Figures and Tables

**Table 1:** Distribution of sociodemographic variables (numbers and percentages).

Variables		Number of people (n)	(%*)
Sex	Female	201	49.3
	Male	207	50.7
Age	18–20 years old	135	33.1
	21–23 years old	70	17.2
	24–26 years old	59	14.5
	27–29 years old	77	18.9
	29 years and above	67	16.4
Educational status	Elementary education	20	4.9
	High school and equivalent	71	17.4
	Degree	250	61.3
	Postgraduate	67	16.4
Event type	Running	296	72.5
	Throwing	46	11.3
	Jumping	66	16.2
Years actively involved in sports	1–3 years	39	9.6
	3–5 years	52	12.7
	5–7 years	93	22.8
	7–10 years	109	26.7
	10 years and above	115	28.2
Taking part in the national team	Yes	177	43.4
	No	231	56.6
How many times have you played in the national team?	0	231	56.6
	1–10	107	26.2
	10–20	55	13.5
	20–30	15	3.7
How important is sports success to you?	Slightly important	11	2.7
	Important	82	20.1
	Very important	315	77.2

* Column percent

**Table 2:** Differences between general total and subdimension scores of the SSSMS and SRQ by sex.

Scale/subscale	Sex	N	Mean score	SD (±)	p
Power display motive	Female	201	35.52	4.368	0.161
	Male	207	36.11	4.105	
Success approach motive	Female	201	61.18	11.042	0.004*
	Male	207	64.00	8.220	
Failure avoidance motive	Female	201	36.39	6.193	0.003*
	Male	207	34.38	7.176	
Grand total	Female	201	133.09	19.151	0.425
	Male	207	134.48	15.870	
Clothing and appearance	Female	201	16.07	5.947	0.475
	Male	207	16.56	7.688	
Objects considered lucky	Female	201	14.60	5.560	0.317
	Male	207	14.05	5.495	
Prior to the game/match	Female	201	23.92	7.402	0.011*
	Male	207	25.86	7.980	
During the game/match	Female	201	2.14	1.282	0.012*
	Male	204	2.49	1.447	
Team behaviors	Female	201	3.10	1.418	0.579
	Male	207	3.18	1.465	
Praying	Female	201	8.35	2.152	0.474
	Male	207	8.20	2.220	
Grand total	Female	201	68.19	19.466	0.285
	Male	207	70.30	20.201	

* p value < 0.05.

**Table 3:** Difference between total and subdimension scores of SSSMS and SRQ by age.

Scale/subscale	Age groups	N	Mean score	SD (±)	p
Power display motive	18–20 years old	135	34.39	4.107	<0.001*
	21–23 years old	70	35.16	5.591	
	24–26 years old	59	37.95	3.288	
	27–29 years old	77	37.35	4.016	
	29 years and above	67	35.73	2.129	
Success approach motive	18–20 years old	135	58.20	7.777	<0.001*
	21–23 years old	70	60.13	13.234	
	24–26 years old	59	61.95	9.879	
	27–29 years old	77	66.90	7.014	
	29 years and above	67	69.73	4.968	
Failure avoidance motive	18–20 years old	135	34.99	5.478	0.435
	21–23 years old	70	35.40	8.632	
	24–26 years old	59	36.08	5.286	
	27–29 years old	77	36.29	7.052	
	29 years and above	67	34.42	7.742	
Grand total	18–20 years old	135	127.59	13.490	<0.001*
	21–23 years old	70	130.69	25.442	
	24–26 years old	59	135.98	16.348	
	27–29 years old	77	140.53	15.086	
	29 years old and above	67	139.88	13.165	
Clothing and appearance	18–20 years old	135	18.93	7.782	<0.001*
	21–23 years old	70	17.63	7.474	
	24–26 years old	59	16.78	4.983	
	27–29 years old	77	13.84	5.294	
	29 years and above	67	12.15	3.986	
Objects considered lucky	18–20 years old	135	14.19	5.257	<0.001*
	21–23 years old	70	16.26	6.670	
	24–26 years old	59	15.63	4.906	
	27–29 years old	77	13.47	4.391	
	29 years and above	67	12.40	5.684	
Before the game and match	18–20 years old	135	24.84	7.752	0.036*
	21–23 years old	70	26.53	8.014	
	24–26 years old	59	25.93	7.615	
	27–29 years old	77	24.79	8.046	
	29 years and above	67	22.57	6.825	
During the game match	18–20 years old	135	2.41	1.340	0.654
	21–23 years old	70	2.43	1.440	
	24–26 years old	59	2.17	1.147	
	27–29 years old	77	2.25	1.359	
	29 years and above	67	2.20	1.595	
Team behaviors	18–20 years old	135	2.92	1.366	0.144
	21–23 years old	70	3.36	1.330	
	24–26 years old	59	3.29	1.509	
	27–29 years old	77	3.05	1.555	
	29 years and above	67	3.33	1.471	
Praying	18–20 years old	135	7.81	2.189	<0.001*
	21–23 years old	70	7.80	2.471	
	24–26 years old	59	8.31	2.581	
	27–29 years old	77	8.78	1.854	
	29 years and above	67	9.09	1.368	
Grand total	18–20 years old	135	71.10	20.999	0.001*
	21–23 years old	70	74.00	21.767	
	24–26 years old	59	72.10	18.536	
	27–29 years old	77	66.18	17.719	
	29 years and above	67	61.64	16.331	

* p value < 0.05.

**Table 4:** Difference between general total and subdimension scores of the SSSMS and SRQ by educational status.

Scale/subscale	Educational status	N	Mean score	SD (±)	p
Power display motive	Primary education	20	33.60	3.530	<0.001*
	High school and equivalent	71	33.93	3.885	
	Degree	250	36.40	4.443	
	Postgraduate	67	36.31	3.168	
Success approach motive	Primary education	20	64.60	4.616	<0.001*
	High school and equivalent	71	56.61	11.153	
	Degree	250	62.39	8.875	
	Postgraduate	67	69.18	8.569	
Failure avoidance motive	Primary education	20	36.40	7.472	<0.001*
	High school and equivalent	71	32.04	6.848	
	Degree	250	35.48	6.501	
	Postgraduate	67	38.18	6.140	
Grand total	Primary education	20	134.60	13.260	<0.001*
	High school and equivalent	71	122.58	17.841	
	Degree	250	134.27	16.677	
	Postgraduate	67	143.67	15.085	
Clothing and appearance	Primary education	20	13.00	4.255	<0.001*
	High school and equivalent	71	19.63	6.823	
	Degree	250	16.34	6.465	
	Postgraduate	67	13.72	7.597	
Objects considered lucky	Primary education	20	10.40	3.979	0.002*
	High school and equivalent	71	15.66	6.000	
	Degree	250	14.42	5.489	
	Postgraduate	67	13.70	4.994	
Before the game and match	Primary education	20	23.00	6.122	0.120
	High school and equivalent	71	24.79	7.071	
	Degree	250	25.53	8.198	
	Postgraduate	67	23.27	6.919	
During the game match	Primary education	20	1.80	.768	<0.001*
	High school and equivalent	71	2.65	1.196	
	Degree	247	2.42	1.454	
	Postgraduate	67	1.73	1.201	
Team behaviors	Primary education	20	2.00	.918	<0.001*
	High school and equivalent	71	2.65	1.097	
	Degree	250	3.26	1.481	
	Postgraduate	67	3.55	1.459	
Praying	Primary education	20	9.40	.821	<0.001*
	High school and equivalent	71	7.11	2.081	
	Degree	250	8.17	2.303	
	Postgraduate	67	9.57	1.033	
Grand total	Primary education	20	59.60	14.547	0.023*
	High school and equivalent	71	72.49	19.414	
	Degree	250	70.12	20.529	
	Postgraduate	67	65.54	17.939	

* p value < 0.05.

**Table 5:** Difference between the general total and subdimension scores of the SSSMS and SRQ by sporting branch.

Scale/subscale	Event type	N	Mean score	SD (±)	p
Power display motive	Running	296	35.94	4.019	0.642
	Throwing	46	35.59	6.166	
	Jumping	66	35.44	3.578	
Success approach motive	Running	296	63.86	8.506	<0.001*
	Throwing	46	60.20	14.593	
	Jumping	66	58.68	9.951	
Failure avoidance motive	Running	296	35.57	6.745	0.572
	Throwing	46	34.50	8.052	
	Jumping	66	35.09	5.950	
Grand total	Running	296	135.36	15.533	0.013*
	Throwing	46	130.28	27.335	
	Jumping	66	129.21	16.617	
Clothing and appearance	Running	296	16.89	7.127	0.002*
	Throwing	46	13.00	5.064	
	Jumping	66	16.08	6.240	
Objects considered lucky	Running	296	14.87	5.593	<0.001*
	Throwing	46	11.15	5.739	
	Jumping	66	14.08	4.237	
Before the game and match	Running	296	25.68	7.795	<0.001*
	Throwing	46	20.52	8.060	
	Jumping	66	24.50	6.249	
During the game match	Running	293	2.45	1.395	<0.001*
	Throwing	46	1.48	.888	
	Jumping	66	2.30	1.381	
Team behaviors	Running	296	3.25	1.439	0.035*
	Throwing	46	2.72	1.486	
	Jumping	66	2.95	1.364	
Praying	Running	296	8.39	2.098	0.251
	Throwing	46	8.00	2.494	
	Jumping	66	7.97	2.327	
Grand total	Running	296	71.50	19.992	<0.001*
	Throwing	46	56.87	19.549	
	Jumping	66	67.88	15.944	

* p value < 0.05.

**Table 6:** Difference between the general total and subdimension scores of the SSSMS and SRQ by years active in sports.

Scale/subscale	Years actively involved in sports	N	Mean score	SD (±)	p
Power display motive	1–3 years	39	33.85	4.277	0.008*
	3–5 years	52	36.15	3.340	
	5–7 years	93	35.52	5.094	
	7–10 years	109	36.67	4.844	
	10 years and above	115	35.77	2.798	
Success approach motive	1–3 years	39	57.31	10.563	<0.001*
	3–5 years	52	60.87	7.685	
	5–7 years	93	59.27	11.800	
	7–10 years	109	63.01	8.942	
	10 years and above	115	67.51	6.843	
Failure avoidance motive	1 to 3 years	39	31.85	5.289	<0.001*
	3–5 years	52	37.65	5.643	
	5–7 years	93	35.27	6.884	
	7–10 years	109	37.36	6.025	
	10 years and above	115	33.73	7.392	
Grand total	1–3 years	39	123.00	13.951	<0.001*
	3–5 years	52	134.67	12.856	
	5–7 years	93	130.05	21.550	
	7–10 years	109	137.04	17.758	
	10 years and above	115	137.01	14.689	
Clothing and appearance	1–3 years	39	16.59	6.069	0.714
	3–5 years	52	17.02	7.737	
	5–7 years	93	16.39	5.616	
	7–10 years	109	16.61	8.254	
	10 years and above	115	15.58	6.256	
Objects considered lucky	1–3 years	39	13.95	4.377	0.165
	3–5 years	52	15.90	7.005	
	5–7 years	93	13.75	4.631	
	7–10 years	109	13.87	5.516	
	10 years and above	115	14.63	5.732	
Before the game and match	1–3 years	39	26.56	6.488	0.467
	3–5 years	52	25.33	8.340	
	5–7 years	93	24.48	7.710	
	7–10 years	109	24.11	8.718	
	10 years and above	115	25.24	6.893	
During the game match	1–3 years	39	2.10	1.165	0.377
	3–5 years	52	2.27	1.285	
	5–7 years	93	2.46	1.522	
	7–10 years	109	2.17	1.302	
	10 years and above	115	2.44	1.426	
Team behaviors	1–3 years	39	2.51	1.393	0.024*
	3–5 years	52	3.04	1.371	
	5–7 years	93	3.20	1.479	
	7–10 years	109	3.10	1.509	
	10 years and above	115	3.38	1.342	
Praying	1–3 years	39	7.79	1.735	0.287
	3–5 years	52	8.02	1.995	
	5 to 7 years	93	8.41	2.529	
	7–10 years	109	8.17	2.466	
	10 years and above	115	8.55	1.768	
Grand total	1–3 years	39	69.51	15.471	0.866
	3–5 years	52	71.58	22.488	
	5–7 years	93	68.70	19.537	
	7–10 years	109	68.03	21.888	
	10 years and above	115	69.76	18.269	

* p value < 0.05.

**Table 7:** Difference between the general total and subdimension scores of the SSSMS and SRQ by national team participation.

Scale/subscale	Taking part in the national team	N	Mean score	SD (±)	p
Power display motive	Yes	177	36.39	4.096	0.017*
	No	231	35.38	4.308	
Success approach motive	Yes	177	66.67	7.990	<0.001*
	No	231	59.49	9.937	
Failure avoidance motive	Yes	177	36.18	6.383	0.036*
	No	231	34.75	7.015	
Grand total Failure avoidance motive	Yes	177	139.24	15.238	<0.001*
	No	231	129.62	18.094	
Clothing and appearance	Yes	177	16.01	7.387	0.418
	No	231	16.56	6.474	
Failure avoidance motive	Yes	177	14.18	6.017	0.649
	No	231	14.43	5.131	
Before the game and match	Yes	177	24.45	7.689	0.303
	No	231	25.25	7.798	
During the game match	Yes	174	2.05	1.291	0.001*
	No	231	2.52	1.408	
Team behaviors	Yes	177	3.20	1.412	0.435
	No	231	3.09	1.464	
Praying	Yes	177	8.30	2.325	0.840
	No	231	8.26	2.077	
Grand total	Yes	177	68.16	20.642	0.326
	No	231	70.11	19.216	

* p value < 0.05.

**Table 8:** Difference between the general total and subdimension scores of the SSSMS and SRQ by the number of national team participations.

Scale/subscale	How many times have you played in the national team?	N	Mean score	SD (±)	p
Power display motive	0	231	35.38	4.308	0.009*
	1–10	107	36.42	4.350	
	10–20	55	36.98	3.664	
	20–30	15	34.00	2.878	
Success approach motive	0	231	59.49	9.937	<0.001*
	1–10	107	66.03	7.343	
	10–20	55	69.27	8.050	
	20–30	15	61.73	9.407	
Failure avoidance motive	0	231	34.75	7.015	<0.001*
	1–10	107	36.18	6.278	
	10–20	55	38.09	5.806	
	20–30	15	29.13	4.033	
Grand total	0	231	129.62	18.094	<0.001*
	1–10	107	138.63	15.334	
	10–20	55	144.35	13.613	
	20–30	15	124.87	9.855	
Clothing and appearance	0	231	16.56	6.474	0.045*
	1–10	107	15.03	5.468	
	10–20	55	18.09	10.002	
	20–30	15	15.33	6.997	
Objects considered lucky	0	231	14.43	5.131	0.082
	1–10	107	13.73	6.111	
	10–20	55	15.62	6.096	
	20–30	15	12.13	3.852	
Before the game and match	0	231	25.25	7.798	0.017*
	1–10	107	24.00	7.094	
	10–20	55	26.53	8.867	
	20–30	15	20.07	4.464	
During the game match	0	231	2.08	1.408	0.002*
	1–10	107	2.12	1.351	
	10–20	52	2.52	1.296	
	20–30	15	1.47	.516	
Team behaviors	0	231	3.09	1.464	0.001*
	1–10	107	3.17	1.417	
	10–20	55	3.60	1.241	
	20–30	15	2.00	1.309	
Praying	0	231	8.26	2.077	0.129
	1–10	107	8.25	2.419	
	10–20	55	8.69	2.133	
	20–30	15	7.20	2.042	
Grand total	0	231	70.11	19.216	0.010*
	1–10	107	66.30	18.743	
	10–20	55	74.49	24.294	
	20–30	15	58.20	11.409	

* p value < 0.05.

**Table 9:** Difference between the general total and subdimension scores of the SSSMS and SRQ by importance placed in sporting success.

Scale/subscale	How important is sports success to you?	N	Mean score	SD (±)	p
Power display motive	Slightly important	11	26.27	6.559	<0.001*
	Important	82	35.79	3.874	
	Very important	315	36.16	3.844	
Success approach motive	Slightly important	11	28.91	7.635	<0.001*
	Important	82	61.04	8.114	
	Very important	315	64.19	7.923	
Failure avoidance motive	Slightly important	11	23.82	11.241	<0.001*
	Important	82	34.63	5.706	
	Very important	315	35.97	6.476	
Grand total	Slightly important	11	79.00	24.731	<0.001*
	Important	82	131.46	14.821	
	Very important	315	136.31	14.503	
Clothing and appearance	Slightly important	11	13.09	4.036	0.007*
	Important	82	18.26	7.550	
	Very important	315	15.93	6.683	
Objects considered lucky	Slightly important	11	9.45	2.770	<0.001*
	Important	82	12.95	3.934	
	Very important	315	14.85	5.810	
Before the game and match	Slightly important	11	17.09	5.683	0.003*
	Important	82	25.28	7.686	
	Very important	315	25.08	7.708	
During the game match	Slightly important	11	1.27	.467	<0.001*
	Important	82	2.78	1.305	
	Very important	315	2.23	1.381	
Team behaviors	Slightly important	11	1.27	.467	<0.001*
	Important	82	2.83	1.294	
	Very important	315	3.29	1.444	
Praying	Slightly important	11	4.73	2.724	<0.001*
	Important	82	7.35	2.696	
	Very important	315	8.64	1.812	
Grand total	Slightly important	11	46.91	15.043	<0.001*
	Important	82	69.45	17.384	
	Very important	315	69.99	20.182	

* p value < 0.05.

**Table 10:** Relationships between the total and subdimension scores of the SSSMS and SRQ.

Scale/subscale	Power display motive	Success approach motive	Failure avoidance motive	Grand total
Clothing and appearance	*R value*			
	−0.020	−0.142*	− 0.002	− 0.085
Objects considered lucky	0.151*	0.055	0.103*	0.107*
Before the game and match	0.098*	0.094	0.068	0.102*
During the game match	−0.103*	0.001	−0.180***	−0.094
Team behaviors	0.150*	0.173***	0.125*	0.181***
Praying	0.300***	0.446***	0.464*	0.501***
Grand total	0.110*	0.064	0.102*	0.102*

* p value < 0.05,

*** p value < 0.01.

## Data Availability

The data underlying this article can be obtained from the corresponding author upon reasonable request.
